# DNA-PK-mediated phosphorylation of EZH2 regulates the DNA damage-induced apoptosis to maintain T-cell genomic integrity

**DOI:** 10.1038/cddis.2016.198

**Published:** 2016-07-28

**Authors:** Y Wang, H Sun, J Wang, H Wang, L Meng, C Xu, M Jin, B Wang, Y Zhang, Y Zhang, T Zhu

**Affiliations:** 1 Shanghai Public Health Clinical Center and Shanghai Key Laboratory of Organ Transplantation, Zhongshan Hospital, Fudan University, Shanghai 200032, China; 2 Key Laboratory of Stem Cell Biology, Institute of Health Sciences, Shanghai Institutes for Biological Sciences, Chinese Academy of Sciences & Shanghai Jiao Tong University School of Medicine (SJTUSM) and Shanghai Institute of Immunology, Institutes of Medical Sciences, SJTUSM, Shanghai 200031, China; 3 Department of Microbiology and Immunology, Fels Institute for Cancer Research and Molecular Biology, Temple University, Philadelphia, PA 19104, USA

## Abstract

EZH2 is a histone methyltransferase whose functions in stem cells and tumor cells are well established. Accumulating evidence shows that EZH2 has critical roles in T cells and could be a promising therapeutic target for several immune diseases. To further reveal the novel functions of EZH2 in human T cells, protein co-immunoprecipitation combined mass spectrometry was conducted and several previous unknown EZH2-interacting proteins were identified. Of them, we focused on a DNA damage responsive protein, Ku80, because of the limited knowledge regarding EZH2 in the DNA damage response. Then, we demonstrated that instead of being methylated by EZH2, Ku80 bridges the interaction between the DNA-dependent protein kinase (DNA-PK) complex and EZH2, thus facilitating EZH2 phosphorylation. Moreover, EZH2 histone methyltransferase activity was enhanced when Ku80 was knocked down or DNA-PK activity was inhibited, suggesting DNA-PK-mediated EZH2 phosphorylation impairs EZH2 histone methyltransferase activity. On the other hand, EZH2 inhibition increased the DNA damage level at the late phase of T-cell activation, suggesting EZH2 involved in genomic integrity maintenance. In conclusion, our study is the first to demonstrate that EZH2 is phosphorylated by the DNA damage responsive complex DNA-PK and regulates DNA damage-mediated T-cell apoptosis, which reveals a novel functional crosstalk between epigenetic regulation and genomic integrity.

The elimination of expanded T cells and the regulation of T-cell apoptosis in the late phase of the immune response are crucial for maintaining immune homeostasis.^1^ In recent years, an understanding of how the DNA damage response contributes to the regulation of T-cell fate in the immune response has emerged. In response to DNA damage occurring during the inflammatory response, cells initiate DNA repair pathways that are required for host cell survival. If the damage is too severe, cell cycle arrest/apoptosis is initiated.^2^ Lymphocytes are particularly susceptible to DNA damage-induced apoptosis; it has been suggested that this sensitivity serves as a fail-safe mechanism to counter these cells' intrinsic high potential for mutation and clonal expansion. However, the regulatory network of DNA damage-induced apoptosis is not yet completely understood.

Polycomb repressive complex 2 (PRC2) mediates gene silencing by catalyzing the tri-methylation of lysine 27 on histone H3 (H3K27me3) within the gene promoter region. PRC2 controls normal stem cell differentiation and is associated with many malignant tumors.^3^ EZH2, the catalytic subunit of PRC2, is an essential epigenetic regulator of multiple cellular events. Interestingly, PRC2 components have recently been reported to be recruited to DNA damage sites, thus suggesting that EZH2 may be involved in DNA damage response mechanisms.^4, 5, 6, 7^ The roles of EZH2 in governing T-cell survival have been noted by several groups. EZH2 has been shown to have a non-redundant role in T helper (Th)-cell lineage survival, and EZH2 deficiency accelerates effector Th-cell death via death receptor-mediated extrinsic and intrinsic apoptotic pathways.^8^ We have also identified a defect in Bim expression that rescues EZH2-mediated cell death in a graft-versus-host disease mouse model, thus providing a different mechanism.^9^ Furthermore, a recent study has revealed a non-redundant and cell-intrinsic requirement for EZH2 in both regulatory T-cell differentiation and effector T-cell expansion.^10^ Given the diversity of mechanisms by which EZH2 regulates T-cell apoptosis, further exploration is needed.

During DNA repair, a protein kinase, DNA-dependent protein kinase (DNA-PK), functions as a sensor of DNA double-strand breaks (DSBs) and is involved in the non-homologous end-joining (NHEJ) DNA repair pathway.^11^ Once DNA damage is present, the DNA-PK catalytic subunit (DNA-PKcs) is recruited to DNA lesion sites and promotes DNA repair by mediating the phosphorylation of downstream proteins.^12, 13^ The regulatory subunit of DNA-PK, Ku80, together with Ku70, functions as a bridge between the kinase and its substrates and mediates the phosphorylation of many proteins, such as p53, HSP90, TFIID, and c-Jun.^12, 14, 15^ Accumulating evidence indicates that the activity and stability of EZH2 are regulated by posttranslational modifications that are critical for the biological function of PRC2, especially phosphorylation.^16^ However, whether the exact mechanism and function of PRC2 at sites of DSBs correlate with the phosphorylase kinase DNA-PK is still unknown.

We have previously shown that EZH2 has critical roles in regulating the T-cell response in several immune diseases.^9, 17, 18^ Given that EZH2's function and target genes largely depend on its interacting proteins, we sought to reveal a new EZH2 regulatory pathway by identifying new EZH2-interacting proteins in T cells, in hopes of facilitating the development of new drug targets for treating immune diseases. We investigated the function and mechanism of EZH2 in T-cell apoptosis. Using co-immunoprecipitation (Co-IP) coupled mass spectrometry (MS), we found that the NHEJ-related protein Ku80 directly interacts with EZH2 and regulates its methyltransferase activity. Furthermore, we demonstrated that Ku80 bridges EZH2 to DNA-PK complexes, thus facilitating EZH2 phosphorylation and resulting in suppression of EZH2 histone methyltransferase activity and upregulation of EZH2 target genes accordingly. Finally, we demonstrated that inhibition of EZH2 increases the DNA damage level in T cells, a result suggesting that EZH2 might participate in maintaining DNA integrity during the T-cell response. Thus, our work reveals a new mechanism by which DNA damage regulates activated T-cell apoptosis in humans.

## Results

### Identification of EZH2-interacting proteins by MS

**Figure 1:**
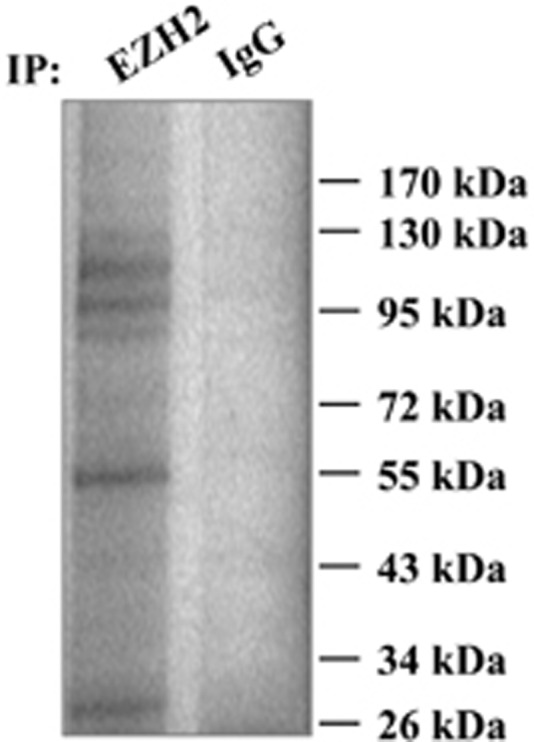
Identification of EZH2-interacting proteins. Immunoprecipitation was performed with anti-EZH2 antibodies on samples from Jurkat T cells. The gels were stained with Coomassie blue

Previous studies have demonstrated that the functions of EZH2 are dependent on its interacting proteins, such as AKT, AP-1, and CDK.^19, 20, 21^ To reveal a novel EZH2-regulation pathway in human T cells, we used protein Co-IP to identify new EZH2-interacting proteins. The nuclear protein fraction obtained from Jurkat T cells was used in this study. Antibodies were pre-cross-linked with magnetic beads to prevent the contamination of antibody chains. As shown in Figure 1 and Table 1, PRC2 components, including EED, SUZ12, and RBBP4, were co-precipitated with anti-EZH2 antibody, thus confirming that the Co-IP system was effective. In addition to the proteins that have been reported to interact with EZH2, we also identified several previously uncharacterized proteins, which could be classified into the following three classes: first, rRNA or pre-mRNA processing proteins, such as Nucleolar RNA helicase 2, Nucleosome assembly protein 1-like 1, Nucleolar protein 56/58, Ribosomal RNA methyltransferase NOP2, and Nucleolin; second, DNA replication-related proteins, such as DNA topoisomerase 1 and the DNA replication licensing factor MCM3/5; third, cellular stress–response proteins, such as heat shock protein 70/90 and X-ray repair cross-complementing protein 5, which is also known as Ku80.

The protein Co-IP data indicated that EZH2 might participate in RNA processing, DNA replication, and the cellular stress–response. Among these proteins, we were interested in the protein Ku80, a DNA damage response protein that has important roles in T-cell development.^22^ We speculated that the interaction between EZH2 and Ku80 might represent an undiscovered mechanism in T cells.

### EZH2 directly interacts with Ku80

**Figure 2:**
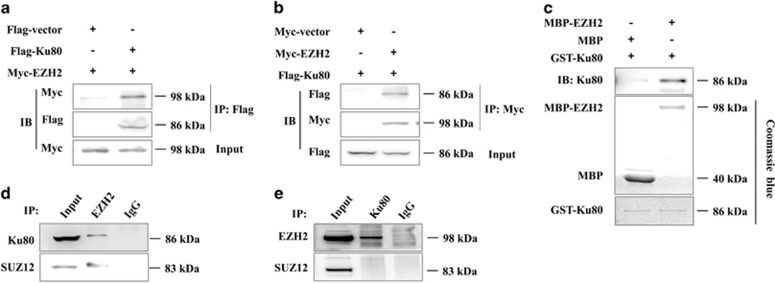
EZH2 directly interacts with Ku80. (**a** and **b**) 293 T cells transfected with plasmids encoding Flag-Ku80 and Myc-EZH2 were immunoprecipitated with anti-Myc or anti-Flag antibodies. The precipitates were analyzed by immunoblotting. (**c**) GST pull-down assays were performed between EZH2 and Ku80. Pulled down samples were subjected to immunoblotting with anti-Ku80 and Coomassie blue staining. (**d** and **e**) Jurkat T cells were collected, and cell lysate was used for immunoprecipitation by anti-EZH2 or anti-Ku80 antibodies. The precipitates were analyzed by immunoblotting

Ku80 is an important component of the DNA-PK complex, which has a critical role in DNA DSB repair and V(D)J gene rearrangement of T cells.^14, 22^ To confirm the interaction between EZH2 and Ku80, we performed reciprocal Co-IP experiments. Plasmids encoding Myc-EZH2 and Flag-Ku80 were co-transfected into 293 T cells, and this was followed by precipitation with Myc- and Flag-specific antibodies. We found that EZH2 and Ku80 were precipitated by each other (Figures 2a and b). To assess whether EZH2 interacts with Ku80 directly, MBP-tagged EZH2 and GST-tagged Ku80 proteins were separately produced with an *Escherichia coli* expression system and subjected to *in vitro* pull-down assays. As shown in Figure 2c, Ku80 protein was co-captured with MBP-EZH2 fusion protein by anti-MBP beads, thus suggesting that EZH2 directly binds to Ku80 in the absence of other PRC2 components. To further validate that the interaction between EZH2 and Ku80 occurs under native conditions, we performed a native Co-IP experiment using Jurkat T cells. SUZ12, a PRC2 component and a known EZH2-binding protein, was used as a positive control. The results indicated that both SUZ12 and Ku80 were co-precipitated by anti-EZH2 antibody. Interestingly, only EZH2 was co-precipitated by anti-Ku80 antibody, thus indicating that the interaction between EZH2 and Ku80 indeed existed *in vivo*, which could occur independently of the PRC2 complex (Figures 2d and e).

### EZH2 cannot methylate Ku80

**Figure 3:**
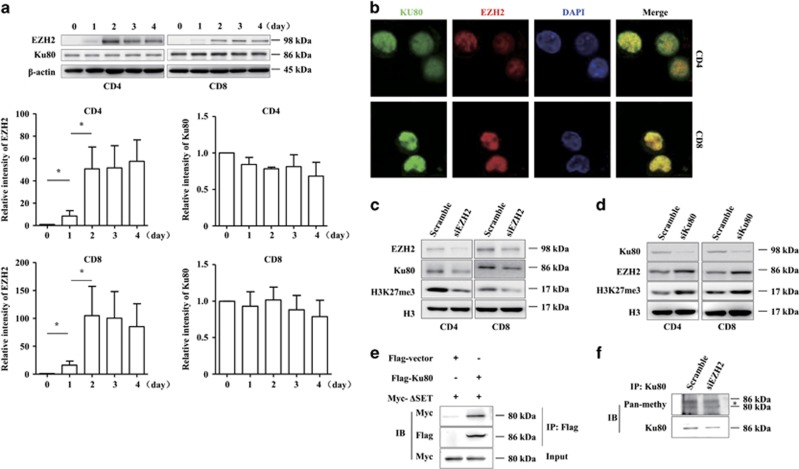
EZH2 cannot methylate Ku80. (**a**) Purified CD4^+^and CD8^+^T cells isolated from PBMC were stimulated with anti-CD3/CD28 antibodies plus IL-2. Cells were collected at different time points for immunoblotting. The band densitometry was quantified using ImageJ software. The quantitative data were calculated from three independent experiments, and were shown the mean±S.E.M. *P<0.05. (**b**) CD4^+^and CD8^+^T cells were stimulated with anti-CD3/CD28 antibodies plus IL-2 for 72 h and then collected for immunostaining analysis. (**c** and **d**) CD4^+^and CD8^+^T cells were stimulated with anti-CD3/CD28 antibodies plus IL-2 for 72 h. Cells were transfected by electroporation with *Ku80* and *EZH2* siRNA or control siRNA and harvested after 48 h. The cell lysates were subjected to immunoblotting. (**e**) 293 T cells transfected with plasmids encoding Flag-Ku80 and SET domain-truncated EZH2 (ΔSET) with Myc-tag were immunoprecipitated with anti-Flag antibody, and the precipitates were subjected to immunoblotting. (**f**) Jurkat T cells were transfected by electroporation with *EZH2* siRNA or control siRNA and harvested after 48 h. The cells were immunoprecipitated with anti-Ku80 antibody, and the precipitates were subjected to immunoblotting. *Represents a non-specific band at~80 kDa

To better understand the functional cross-talk between EZH2 and Ku80, we examined their expression patterns in human primary T cells. Upon T-cell receptor (TCR) activation, EZH2 expression markedly increased in proliferating T cells compared with unstimulated T cells (Figure 3a). As a comparison, Ku80 was constitutively expressed in unstimulated T cells and was not dramatically upregulated in T cells upon TCR activation (Figure 3a). Previous studies have shown that EZH2 and Ku80 are present in both the cytoplasm and nucleus.^23, 24^ To determine their cellular location in TCR-activated T cells, confocal microscopy assay was performed. As shown in Figure 3b, EZH2 and Ku80 colocalized only in the nucleus. To investigate the effect of the EZH2-Ku80 interaction, loss-of-function analysis was performed in human TCR-activated T cells. When EZH2 was knocked down with an EZH2-specific siRNA, the levels of both Ku80 and H3K27me3 markedly decreased (Figure 3c). Interestingly, Ku80 knockdown increased mRNA levels of EZH2, suggesting that Ku80 could affect EZH2 expression at the transcription level (Supplementary Figure S1). In contrast, Ku80 knockdown increased EZH2 and H3K27me3 levels (Figure 3d), a result reflecting the functional cross-talk between EZH2 and Ku80. In addition, posttranslational modification of Ku80 has been shown to be important for regulating its DNA-binding activity.^15^ Therefore, we asked whether Ku80 is an EZH2 substrate. The methyltransferase activity of EZH2 depends on the SET domain. If EZH2 can methylate Ku80, the SET domain would be necessary for their interaction. Therefore, we constructed a truncated EZH2 mutant by deleting the SET domain (referred to as ΔSET) and examined its interaction with Ku80. Co-IP analysis showed that ΔSET was capable of binding to Ku80 (Figure 3e). We further immunoprecipitated Ku80 from Jurkat T cells and then detected methylated Ku80 with an anti-pan-methyl antibody. Methylated Ku80 was detected in lymphocytes, thus suggesting that this methylation was independent of EZH2 (Figure 3f). These data showed that Ku80 is not a methyltransferase substrate of EZH2.

### DNA-PKcs phosphorylates EZH2 *in vitro*

**Figure 4:**
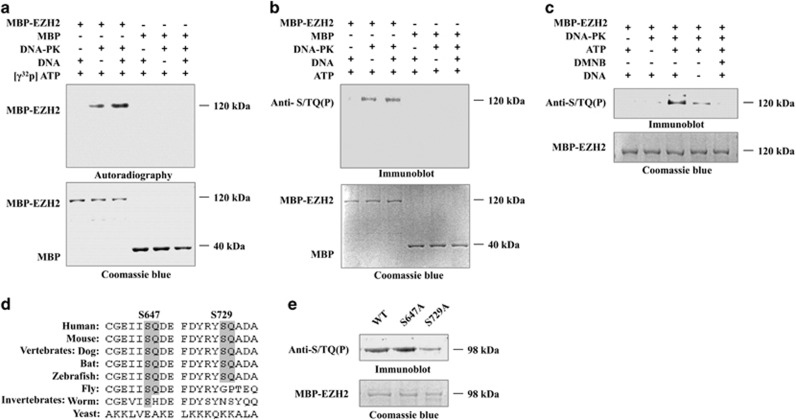
DNA-PKcs phosphorylates EZH2 *in vitro*. (**a**) An *in vitro* reaction system was set up in 50 *μ*l of reaction buffer containing 20 units of DNA-PK complex, 200 *μ*g MBP-EZH2, 200 *μ*M ATP, 5* μ*Ci (γ-^32^P) ATP, 4 *μ*g BSA, and 10 *μ*g/ml calf thymus DNA. The mixture was collected after a 15 min incubation at 37 °C, the signal was detected by autoradiography, and a parallel gel was stained with Coomassie blue. (**b**) ATP without (*γ*-^32^P) was added to the reaction mixture. EZH2 phosphorylation was analyzed by immunoblotting and Coomassie blue staining. (**c**) DMNB (5 *μ*M) was added to the reaction mixture. EZH2 phosphorylation was analyzed by immunoblotting and Coomassie blue staining. (**d**) Schematic alignment of EZH2 S/TQ sites in different species. (**e**) S647A and S729A mutants were generated and subjected to kinase assays. EZH2 phosphorylation was analyzed by immunoblotting and Coomassie blue staining

Ku80 is a multifunctional protein with high DNA-binding activity; this protein is capable of binding different types of DNA structures, including nicks, gaps, and hairpins, as well as the ends of telomeres.^14^ Once bound to DNA, Ku80 promotes the assembly of the DNA-PK complex, which includes DNA-PKcs, Ku70, and Ku80, and then Ku80 and Ku70 serve as a bridge between DNA-PKcs and its substrates, thus facilitating substrate phosphorylation. Given the direct interaction between EZH2 and Ku80, we therefore asked whether EZH2 is actually the substrate of the DNA-PK complex. To validate our hypothesis, an *in vitro* kinase assay was performed. MBP-EZH2 was purified and added together with DNA-PK and (*γ*-^32^P) ATP. As shown in Figure 4a, the DNA-PK complex phosphorylated EZH2 *in vitro*. Previous studies have demonstrated that the DNA-PK complex recognizes S/TQ sites in its substrates' sequences.^25^ To identify whether DNA-PK phosphorylated EZH2 at S/TQ residues, we used an anti-S/TQ (P) antibody to detect the phosphorylation signals. As expected, we detected a phosphorylation signal, thus suggesting that the EZH2 phosphorylation site should be a conserved S/TQ motif (Figure 4b). 4,5-Dimethoxy-2-nitrobenzaldehyde (DMNB) is a DNA-PK complex inhibitor that specifically inhibits the phosphorylation reaction mediated by DNA-PK.^26^ After DMNB was added, EZH2 phosphorylation was abolished, a result further confirming that EZH2 is indeed the substrate of the DNA-PK complex (Figure 4c). To further identify the phosphorylation sites of EZH2, we performed site-directed mutagenesis of EZH2. Sequence alignment indicated that two candidate EZH2 S/TQ sites (S647 and S729) are highly conserved in vertebrates (Figure 4d). Two EZH2 mutants were generated by replacing serine 647 and serine 729 with alanine (referred to as S647A and S729A, respectively) and were subjected to kinase assays. As shown in Figure 4e, the phosphorylation signal was markedly decreased in S729A, thus suggesting that DNA-PK phosphorylates EZH2 at S729.

### DNA-PK inhibits EZH2 histone methyltransferase activity in human CD8^+^T cells

**Figure 5:**
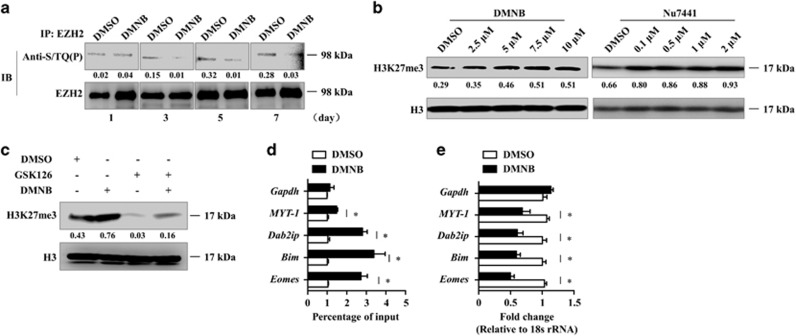
DNA-PK regulates EZH2 HMT activity in human CD8^+^ T cells. (**a**) Purified CD8^+^T cells isolated from PBMCs were stimulated with anti-CD3/CD28 antibodies plus IL-2. At different time points, cells subjected to DMSO or 5 *μ*M DMNB treatment were immunoprecipitated with anti-EZH2 antibody, and this was followed by immunoblotting. (**b**) CD8^+^T cells were stimulated with anti-CD3/CD28 antibodies in the presence of different doses of DMNB and NU7441. Seventy-two hours later, cells were collected for immunoblotting. (**c**) CD8 ^+^T cells were stimulated with anti-CD3/CD28 antibodies in the presence or absence of GSK126 (2 *μ*M) and DMNB (5 *μ*M). Seventy-two hours later, cells were collected for immunoblotting. (**d**) CD8^+^T cells treated with 5 *μ*M DMNB were collected for ChIP assays against H3K27me3 or IgG control. Total input DNA before immunoprecipitation was used for normalization of data. The graph shows the relative amount of H3K27me3 and IgG at the regions of *Eomes*, *Bim*, *Dab2ip*, *MYT-1*, and *Gapdh*. The results are the mean±S.E.M. from three independent experiments. *P<0.05. (**e**) Total RNA was isolated from CD8 ^+^T cells treated with 5 *μ*M DMNB on day 3 post stimulation, and gene expression was determined by real-time PCR analysis. The results are the mean±S.E.M. from three independent experiments. *P<0.05

DNA-PK has critical roles in the DNA damage response by substrate phosphorylation.^4, 5^ To emphasize the significance of DNA-PK mediated EZH2 phosphorylation, functional analysis was performed on human CD8^+^T cells. We immunoprecipitated EZH2 from CD8^+^T cells at 1, 3, 5, and 7 days after activation. We found that the level of phosphorylated EZH2 was significantly increased in TCR-activated CD8^+^T cells at 5 and 7 days after activation, whereas adding DNA-PK inhibitor moderately reduced this level (Figure 5a). We next measured the effects of inhibiting DNA-PK on EZH2 methyltransferase activity by examining the levels of H3K27me3. The addition of DMNB dose-dependently increased the expression of cellular H3K27me3 in activated CD8^+^T cells. Similar results were obtained with another DNA-PK inhibitor, NU7441^27^ (Figure 5b). GSK126 is a specific EZH2 inhibitor.^28^ As expected, GSK126 treatment significantly decreased H3K27me3 levels in T cells, and its effect was reversed when DNA-PK inhibitor was added (Figure 5c). These data suggest that DNA-PK has an important role in regulating the methyltransferase activity of EZH2 in T cells. Real-time PCR analysis confirmed that the addition of DMNB resulted in the repression of *Eomes*, *Bim*, *Dab2ip*, and *MYT-1* (Figures 5d and e), which are known EZH2 target genes that are expressed in activated CD8^+^T cells.^29, 30^ Thus, the DNA-PK complex might regulate EZH2 methyltransferase activity by phosphorylating EZH2.

### EZH2 inhibition increases the risk of DNA damage-mediated T-cell apoptosis

**Figure 6:**
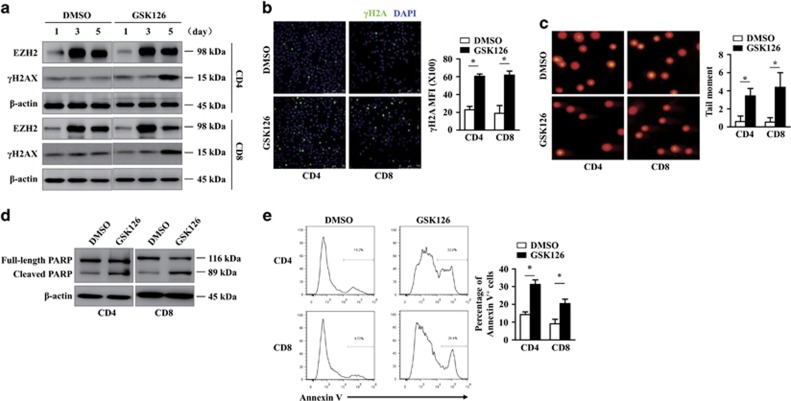
EZH2 inhibition increases the risk of DNA damage-mediated T-cell apoptosis. Purified CD4^+^and CD8^+^T cells isolated from PBMCs were stimulated with anti-CD3/CD28 antibodies plus IL-2 in the presence or absence of GSK126 (2 *μ*M). (**a**) Cells were collected at different time points as indicated, and protein expression was determined by immunoblotting. (**b**) Cells were collected on day 5, and γH2AX expression was assessed by immunofluorescence staining. The graph shows the mean fluorescence intensity of cells with *γ*H2AX foci among at least 100 cells. The data represent at least three independent experiments. *P<0.05. (**c**) Cells were collected on day 5. A representative image of comet assay results is shown. The tail moments were measured with OPENCOMET. *P<0.05. (**d**) Cells were collected on day 5 and cell lysates were collected to determine protein expression using immunoblotting. (**e**) The fraction of annexin V-positive donor-derived T cells collected on day 5 was analyzed by flow cytometry. The data represent at least three independent experiments. *P<0.05

DNA-PK has critical roles in the DNA damage response, and PRC2 components have recently been reported to be recruited to DNA damage sites.^5^ We therefore asked whether EZH2 has a role in DNA damage-mediated T-cell apoptosis. The addition of GSK126 to cultured human CD4^+^and CD8^+^T cells activated by anti-CD3/CD28 antibodies markedly increased cellular γH2AX expression on day 5 (Figures 6a and b). Comet assays further confirmed the increase of DNA fragments in activated T cells treated with GSK126 (Figure 6c). This result suggests that inhibiting EZH2-catalyzed H3K27me3 may affect genomic stability in activated T cells. It has been shown that DNA damage induces cell apoptosis. Significant increases in the levels of cleaved PARP and in the fraction of annexin V-positive cells were observed in the GSK126-treated group, thus suggesting that EZH2 inhibition increases apoptosis (Figures 6d and e). To confirm the effect of EZH2 on T-cell apoptosis, we used etoposide as the external source of DNA damage, a DNA-damaging agent that has previously been reported to induce DNA DSBs and to upregulate several DNA damage markers.^31^ Immunoblot analyses revealed the accumulation of cleaved PARP after etoposide treatment. Furthermore, co-incubation with GSK126 and etoposide resulted in statistically significant increase in activated T-cell apoptosis (Supplementary Figure S2). These results suggest that EZH2 inhibition might increase the risk of DNA damage-mediated apoptosis during the T-cell response. Recently, DNA damage caused by exposure to DNA-damaging agents has been shown to induce autophagy, a cellular catabolic process that maintains cellular homeostasis.^32^ Inhibition of EZH2 in T cells substantially increased autophagy induction, as measured by LC3A II conversion, which is a common readout for autophagy,^33^ upregulation of BECN1, an essential molecule in autophagosome formation^34^ (Supplementary Figure S3A) and puncta structure formation (Supplementary Figure S3B). Besides, EZH2 inhibitor treatment-induced increase of the puncta structure formation was enhanced by the lysosomal inhibitor chloroquine^35^ treatment, which provided an estimation of the autophagic flux (Supplementary Figure S3B). Furthermore, *BECN1* knockdown with an *BECN1*-specific siRNA increased the cleaved PARP in T cells treated with GSK126, suggesting the protective role of autophagy in EZH2 inhibition-mediatated apoptosis (Supplementary Figure S3C).

## Discussion

EZH2 is involved in a variety of cellular activities, such as cell proliferation, migration, invasion, stem cell fate decision, and tumorigenesis.^3, 36^ Recently, research interests have shifted toward the role of EZH2 in regulation of gene repression and its cellular context in immune cells; however, its roles in maintaining T-cell genomic integrity after activation are less clear. Here, we analyzed potential EZH2-interacting proteins by MS, and we found that EZH2 can directly interact with a DNA damage responsive protein Ku80. The interaction between EZH2 and Ku80 may represent a new mechanism regulating cell survival. We demonstrated that Ku80 bridges the interaction between DNA-PK complexes, and resulting in EZH2 phosphorylation at S729. Consequently, the methyltransferase activity of phosphorylated EZH2 decreases, thus resulting in the dynamic regulation of the cellular H3K27me3 level. Given our observation that inhibition of EZH2 increased the risk of DNA damage in activated T cells during proliferation, we suggest that DNA-PK-mediated EZH2 phosphorylation may represent a novel pathway regulating the cross-talk between DNA damage and epigenetic regulation.

EZH2 has a central role as a catalytic enzyme involved in deposition of H3K27me3, which silences target gene transcription. EZH2 has been found to be enriched in chromatin damage sites, and EZH2 deficiency accelerates effector Th-cell death via death receptor-mediated extrinsic and intrinsic apoptotic pathways.^8^ Our previous study has also demonstrated that inhibition of EZH2 arrests ongoing graft-versus-host disease by inducing apoptosis in alloreactive T cells and that this induction of apoptosis is largely independent of the proapoptotic protein Bim.^9^ Interestingly, the underlying mechanism remains unclear. However, an H3K27me3-independent mechanism in which EZH2 functions as a co-activator of androgen receptor for gene activation has been reported.^37^ Therefore, further studies are need to illustrate whether phosphorylation of EZH2 at S729 has transcriptional activator function.

Accumulating evidence suggests that EZH2 activity and stability are tightly regulated by multiple posttranslational modifications. AKT-mediated EZH2 phosphorylation at Ser21 decreases H3K27me3 activity, thus resulting in transcriptional activation of a subset of its target genes.^28^ Several studies have demonstrated that CDK1/2 phosphorylates EZH2 at multiple sites, among which Thr 345 phosphorylation is required for the maintenance of H3K27me3 repressive marks at target gene promoters.^38^ JAK2 phosphorylates EZH2 at tyrosine 641 (Y641), which is frequently mutated in B-cell lymphoma, and the stability and activity of the EZH2 Y641 mutant is higher than that of wild-type EZH2.^39^ Here, we found that EZH2 is phosphorylated by DNA-PK and that inhibition of DNA-PK impairs the dynamic H3K27me3 level, thus suggesting that DNA-PK might be able to affect local gene expression by regulating EZH2. As a central integrator of the DNA damage response, DNA-PK is involved in DSB repair and maintains genome stability and integrity. Furthermore, DNA-PK has also been reported to regulate innate immune responses, especially T-cell apoptosis. Integrase inhibitors and interventions directed toward DNA-PK might improve T-cell survival and immune function during human immunodeficiency virus-1 infection.^40^ One DNA-PK inhibitor has been developed as an anti-cancer agent on the basis of its ability to potentiate cell death mediated by chemotherapy- and radiation therapy-induced DSBs, thus suggesting that DNA-PK coupled with small molecule pharmacological inhibitors might be developed as a novel treatment approach for patients with T cell-associated malignancies.

Overexpression and dysregulated of EZH2 are frequently observed in multiple types of cancer. The expression level of EZH2 is also correlated with poor prognosis in patients with lung, breast, and prostate cancer.^41^ EZH2 functions as a tumor suppressor in many types of cancer; however, inactivating mutations of EZH2 have been found in patients with myeloid malignancies, including myelodysplastic syndrome and myeloproliferation neoplasms, and have been associated with poor patient survival.^42, 43^ Mice with conditional deletions of *EZH2* and *TET2* in hematopoietic stem cells develop myelodysplastic syndrome and myeloproliferative neoplasms.^44, 45^ Loss-of-function mutations and deletions of the *EZH2* and *SUZ12* genes have been reported in 25% of T-cell acute lymphoblastic leukemias.^46^ Besides, the conditional deletion of EZH2 in bone marrow cells causes T-cell leukemia.^47^ These results suggest that the oncogenic role of EZH2 in hematological malignancies differs from that in solid tumors. Recently, several highly selective small molecule inhibitors against EZH2, such as GSK126 and EPZ-6438, have been developed. Currently, EPZ-6438 is being tested in clinical trials in patients with B-cell lymphoma (NCT01897571). The elimination of expanded T cells at the end of the immune response is crucial for maintaining homeostasis. In the late phase of the T-cell response, DNA damage increases, and this triggers the so-called damage-induced cell apoptosis or even death and may facilitate the depletion of terminally differentiated T cells. When Ku80 was knocked down or when DNA-PK was inhibited, we found that histone methyltransferase activity of EZH2 was enhanced, thus suggesting that DNA-PK-mediated EZH2 phosphorylation impairs EZH2 histone methyltransferase activity. On the other hand, EZH2 inhibition increased the DNA damage level at the late phase of T-cell activation. Interestingly, our results also indicated that inhibition of EZH2 in activated T cells increased autophagy, which is a highly conserved cellular recycling and maintenance mechanism that maintains cellular homeostasis. Several groups have observed hyper-autophagic phenotypes after antigenic stimulation are associated with defective T-cell survival and clonal expansion.^48^

Inhibition of EZH2 has been shown to lead to cell differentiation or prematuration,^49, 50^ whereas terminal differentiation or senescence is concomitant with increased DNA damage. Here, we found that when the methyltransferase activity of EZH2 was inhibited, the time point of DNA damage was much earlier than that of controls, thus suggesting that the critical roles of EZH2 in regulating T-cell fate. Upon antigen stimulation, T cells undergo rapid expansion and later depletion. How the genome replicates accurately and whether accurate replication is correlated with the later programmed apoptosis is still a mystery. It would be interesting to determine whether DNA damage can also be precisely ‘programmed' during the T-cell response by the epigenetic regulation factor to reduce the risk of inflammation. Our finding confirm a direct correlation between epigenetic regulation and the DNA damage response. Further studies are needed to address the functions of DNA damage, DNA-PK, and the EZH2 axis.

## Materials and Methods

### Cell culture

Jurkat T and 293 T-cell lines were purchased from American Type Culture Collection (Manassas, VA, USA). Human primary peripheral blood mononuclear cells (PBMC) from healthy volunteers were isolated from whole blood samples with Ficoll-Paque Plus (GE Healthcare Bio-Sciences AB, Uppsala, Sweden) using density gradient separation. This study was approved by the Ethics Committee of Shanghai Changhai Hospital. Whole blood for the study was obtained from healthy volunteers who had provided written informed consent. Jurkat T cells and PBMC were cultured in RPMI 1640 growth medium supplemented with penicillin (100 U/ml), streptomycin (100 *μ*g/ml), l-glutamine (2 mM), and 10% fetal bovine serum (FBS). 293 T cells were cultured in DMEM medium supplemented with penicillin (100 U/ml), streptomycin (100 *μ*g/ml), and 10% FBS (All from Life Technologies GmbH, Darmstadt, Germany).

### Primary T-cell isolation and activation

CD4^+^ and CD8^+^T cells were magnetically purified from PBMC according to the manufacturer's recommendations (Miltenyi Biotec, Auburn, CA, USA). The purity of the sorted cells in this study was consistently >95%. The primary T cells were activated in the presence of plate-bound anti-CD3 (2 *μ*g/ml) and soluble anti-CD28 (2 *μ*g/ml) (both from eBioscience, San Diego, CA, USA).

### Reagents and antibodies

All siRNAs were purchased from GenePharma (Shanghai, China). Antibodies against EZH2 (3147), H3K27me3 (9733), *β*-actin (4967), Myc-tag (2278), Flag-tag (2368), SUZ12 (3737), LC3A/B (4108), BECN1 (3495), and phospho-S/T [Q] (2851) were obtained from Cell Signaling Technology (Danvers, MA, USA). Antibodies against Ku80 (PA5-17457) and *γ*H2AX (pS139) (PA5-35464) were obtained from Thermo (Waltham, MA, USA) and Epitomics (Burlingame, CA, USA), respectively. Antibodies against EZH2 (17662) were obtained from Millipore (Billerica, MA, USA). Secondary antibodies used for immunofluorescence and flow cytometry were as follows: Alexa Fluor 488-conjugated goat anti-rabbit (A11070) and Alexa Fluor 594-conjugated goat anti-mouse (A21125) (Invitrogen, Carlsbad, CA, USA). A FITC Annexin V Apoptosis Detection Kit was purchased from BD (556547) (BD Biosciences, Franklin Lakes, NJ, USA). Microbead-conjugated antibodies were obtained from Miltenyi-Biotech (Bergisch Gladbach, Germany), and IL-2 was purchased from PeproTech (Rocky Hill, NJ, USA). DMNB was purchased from Millipore (814335), NU7441 was purchased from Selleck (Shanghai, China), chloroquine and etoposide was purchased from Sigma-Aldrich Co. (St. Louis, MO, USA).

### MBP-EZH2 and GST-Ku80 protein purification and pull-down assay

MBP- and GST-tagged proteins were both expressed in *E. coli* strain BL21 (DE3) (plyS). Cells were harvested by centrifugation and resuspended in 20 ml of lysis buffer (50 mM Tris-HCl (pH 8.0) 500 mM NaCl, 0.22 mg/ml lysozyme, 100 *μ*M PMSF, and 10 mM DTT) and sonicated on ice. The lysate was centrifuged, and the supernatant was mixed with Ni-NTA slurry (Qiagen, Venlo, the Netherlands) and rocked for 60 min at 4 °C. The mixture was poured through a Ni-NTA column and washed with 50 ml of phosphate wash buffer (50 mM Tris-HCl (pH 8.0), 20 mM imidazole, 500 mM NaCl, and 10% glycerol). Purified protein was eluted with elution buffer (500 mM imidazole, 1 M NaCl, 10% glycerol, and 50 mM Tris-HCl (pH 8.0)). The imidazole and excess NaCl were removed by dialysis in buffer (50 mM Tris-HCl, 150 mM NaCl and 10% glycerol).

MBP and MBP-EZH2 proteins were incubated at 4 °C with GST-Ku80 proteins overnight. MBP-tagged proteins were captured by incubation with anti-MBP magnetic beads (New England Biolabs, Ipswich, MA, USA) at 4 °C for 3 h. The bead pellet was washed five times with 500 *μ*l of buffer (50 mM Tris-HCl (pH 7.5), 150 mM NaCl, 1% NP-40, 10% glycerol and 2 mM EDTA). Samples were boiled and subjected to SDS-PAGE.

### *In vitro* kinase assay

Kinase assays were conducted in 50 *μ*l reaction buffer (50 mM HEPES (pH 7.5), 100 mM KCl, 10 mM MgCl, 200 *μ*M EGTA, 100 *μ*M EDTA, and 5 mM DTT) containing 20 units of DNA-PK complex (Promega, Fitchburg, WI, USA), 200 *μ*g MBP-EZH2, 200 *μ*M ATP, 5 *μ*Ci (*γ*-^32^P) ATP, 4 *μ*g BSA, and 10 *μ*g/ml calf thymus DNA. The mixture was incubated at 30 °C for 15 min. The reaction mixture was subjected to SDS-PAGE and subsequently detected by autoradiography.

### Western blot and immunoprecipitation

For western blotting, proteins were extracted with RIPA buffer (Thermo), sonicated briefly and separated by SDS-PAGE. For the Co-IP coupled MS, anti-EZH2 and IgG antibodies were cross-linked with anti-protein A/G magnetic beads (Pierce, Rockford, IL, USA) with disuccinimidyl suberate (Pierce). Cell proteins were extracted with lysis buffer (150 mM NaCl, 1.0% NP-40, and 50 mM Tris-Cl (pH 7.4)). Cross-linked beads were cultured with cell lysate overnight and then washed three times with lysis buffer. Next, the beads were resuspended in RIPA buffer and subjected to SDS-PAGE. After electrophoresis, the gels were stained with Coomassie blue, and bands were cut and analyzed by MS. The densitometry of the bands was quantified using ImageJ software.

### Transfection

Primary T cells and Jurkat T cells were transfected by electroporation using a Human T Cell Nucleofector Kit (Lonza, Basel, Switzerland) according to the manufacturer's protocol. Cells were suspended in Nucleofector Solution to a final concentration of 2.5 × 10^5^ cells/100 *μ*l. The cells were transfected using either 30–300 nM of siRNA or 2 *μ*g of GFP plasmid. In Amaxa certified cuvettes, the cells were nucleofected using the program T-020. The nucleofected cells were transferred to 400 *μ*l of prewarmed medium in 48-well plates and incubated for 4–20 h. Cells were analyzed 4–20 h post transfection. For liposome-mediated transfection, cells were transfected using Lipofectamine 2000 according to the manufacturer's recommendations (Invitrogen, 11668019).

### Confocal microscopy

Cells grown on coverslips were fixed in BD Cytofix/Cytoperm solution (554714) at room temperature for 15 min. These coverslips were incubated with the primary antibody followed by fluorochrome-conjugated secondary antibody before mounting. For fluorescence analysis, cell samples were visualized on an Olympus FluoView confocal microscope with appropriate emission filters (Olympus, Tokyo, Japan).

### Real-time PCR and chromatin immunoprecipitation (CHIP) assay

Total RNA was extracted from T cells at the indicated times and was subsequently reverse-transcribed using a Reverse Transcription System (DRR036A, TaKaRa, Tokyo, Japan). Quantitative PCR was performed using SYBR Green PCR mix (4913914001, Roche, Basel, Switzerland) on an ABI Prism 7900HT Sequence Detection System (Applied Biosystems, Foster City, CA, USA), and 18 s rRNA was used as an internal control to normalize for differences in the amount of total RNA in each sample. CHIP was performed according to the manufacturer's recommendations (Millipore). The primer sequences used for PCR analysis are listed in the Supplementary Table.

### Comet assay

Comet assays were performed as described previously.^51^ Ten microliter aliquots of cell suspension (~15 000 cells) were mixed with 120 *μ*l of low melting point agarose (0.5% in PBS) and added to microscope slides (with frosted ends) that had been covered with a bottom layer of 1.5% agarose. Slides were lysed (pH 10; 4 °C) for at least 1 h. When the comet assay was combined with post-treatment with bacterial FPG protein, the slides were washed three times (for 5 min each) in enzyme buffer (40 mM HEPES, 100 mM KCl, 0.5 mM EDTA, and 0.2 mg/ml BSA (pH 8.0)), covered with 100 *μ*l of either buffer or FPG protein in buffer, sealed with a coverslip and incubated for 30 min at 37 °C. Slides with or without FPG post-treatment were processed using an alkali denaturation (at a pH>13) time of 25 min and electrophoresis (0.86 V/cm) for 25 min. Slides were coded, and images of 100 randomly selected cells stained with ethidium bromide were analyzed from each slide. Measurements were made by image analysis (Comet Assay IV, Perceptive Instruments, Haverhill, UK), and DNA migration was determined by measuring the ‘tail intensity' (% tail DNA).

### Statistical analysis

GraphPad Prism (version 4.0, GraphPad Software, Inc., La Jolla, CA, USA) and SPSS 17.0 (SPSS Inc. Chicago, IL, USA) were used for statistical analyses. Significant differences were evaluated using an independent-samples *t*-test or Wilcoxon rank test. *P*<0.05 was considered to be statistically significant.

## Figures and Tables

**Table 1:** Identification of EZH2-interacting proteins with MS

**Protein name**	**Total spectrum count**	**Percentage sequence coverage**
*PRC2*		
Histone-lysine N-methyltransferase EZH2	81	50.30%
Polycomb protein SUZ12	113	58.50%
Polycomb protein EED	11	22.50%
Histone-binding protein RBBP4	9	15.40%
Metal-response element-binding transcription factor 2	1	4.96%

*Cellular stress response*		
X-ray repair cross-complementing protein 5	27	27.90%
Heat shock protein 90-beta	14	22.50%
Heat shock cognate 70 protein	3	6.38%

*DNA replication*		
DNA topoisomerase 1	23	18.60%
DNA replication licensing factor MCM5	2	4.05%
DNA replication licensing factor MCM3	2	4.60%

*RNA processing*		
Isoform 2 of Nucleolar RNA helicase 2	14	11.20%
Nucleosome assembly protein 1-like 1	20	19.30%
Nucleolar protein 56	5	12.10%
Nucleolar protein 58	2	5.10%
Rbosomal RNA methyltransferase NOP2	11	12.30%
Nucleolin	12	16.60%

## Abbreviations

DSBs: double-strand breaks
PRC2: polycomb repressive complex 2
H3K27me3: tri-methylation of lysine 27 on histone H3
Th: T helper
DNA-PK: DNA-dependent protein kinase
DNA-PKcs: DNA-PK catalytic subunit
NHEJ: non-homologous end-joining
MS: mass spectrometry
Co-IP: co-immunoprecipitation
TCR: T-cell receptor
DMNB: 4,5-dimethoxy-2-nitrobenzaldehyde
PBMCs: peripheral blood mononuclear cells
CHIP: chromatin immunoprecipitation
