# Supplementary material for: DNA-PK-mediated phosphorylation of EZH2 regulates the DNA damage-induced apoptosis to maintain T-cell genomic integrity
Source: Cell Death Dis. 2016 Jul 28;7(7):e2316–. doi: 10.1038/cddis.2016.198 (PMC4973345; doi:10.1038/cddis.2016.198)
Supplement: Supplementary Figure Legends [file cddis2016198x1.docx]

**Supplementary Figure Legends**

**Supplementary Figure S1. Ku80 could affect EZH2 expression at the transcription level (A)** CD4 ^+^ and CD8 ^+^ T cells were stimulated with anti-CD3/CD28 antibodies plus IL-2 for 72 hours. Cells were transfected by electroporation with *Ku80* siRNA or control siRNA and harvested after 48 hours. Total RNA was isolated and gene expression was determined by real-time PCR analysis. The results are the mean ± S.E.M. from three independent experiments. * *P* < 0.05.

**Supplementary Figure S2. DNA damage promotes the T cell apoptosis.** (A) Purified CD4 ^+^ and CD8 ^+^ T cells were stimulated with anti-CD3/CD28 antibodies in the presence or absence of GSK126 (2 μM) and etoposide (50 μM). Twenty-four hours later, cells were collected for western blot analysis.

**Supplementary Figure S3. EZH2 inhibition increase the DNA damage-mediated T cell autophagy.** (A) Purified CD4 ^+^ and CD8 ^+^ T cells isolated from PBMCs were stimulated with anti-CD3/CD28 antibodies plus IL-2 in the presence or absence of GSK126 (2 μM). Cells were collected on day 5, and cell lysates were collected to determine protein expression by western blot analysis. (B) Purified CD4 ^+^ and CD8 ^+^ T cells transfected with LC3A overnight on day 2 post stimulation were cultured in GSK126 (2 μM) for 72 hours with or without chloroquine pretreatment (50 μM, 3 hours), and then fixed. Digital images were captured with confocal microscopy. The data represents at least three independent experiments. * *P* < 0.05 (C) CD4 ^+^ and CD8 ^+^ T cells were stimulated with anti-CD3/CD28 antibodies plus IL-2 for 72 hours. Cells were transfected by electroporation with *BECN1* siRNA or control siRNA and treated with GSK126 (2 μM) for 48 hours. Protein expression was determined by immunoblotting.
