# Supplementary figures and images for: DNA-PK-mediated phosphorylation of EZH2 regulates the DNA damage-induced apoptosis to maintain T-cell genomic integrity
Source: Cell Death Dis. 2016 Jul 28;7(7):e2316–. doi: 10.1038/cddis.2016.198 (PMC4973345; doi:10.1038/cddis.2016.198)

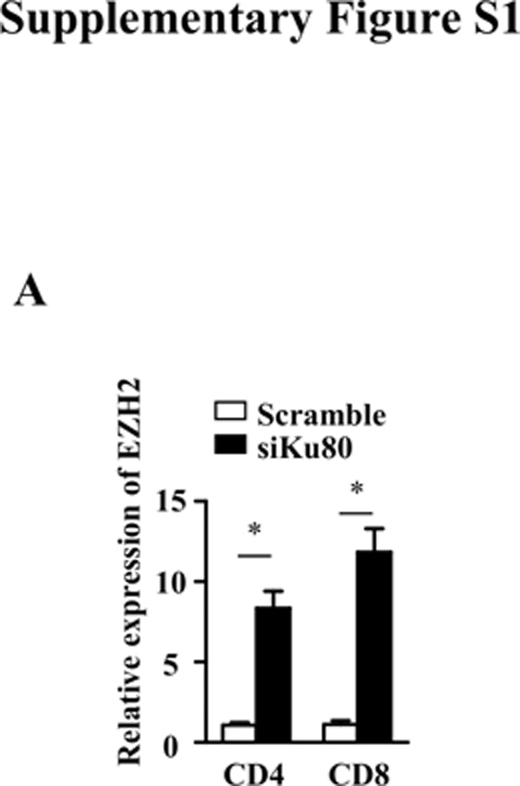

Supplement: Supplementary Figure 1 [file cddis2016198x2.tif]

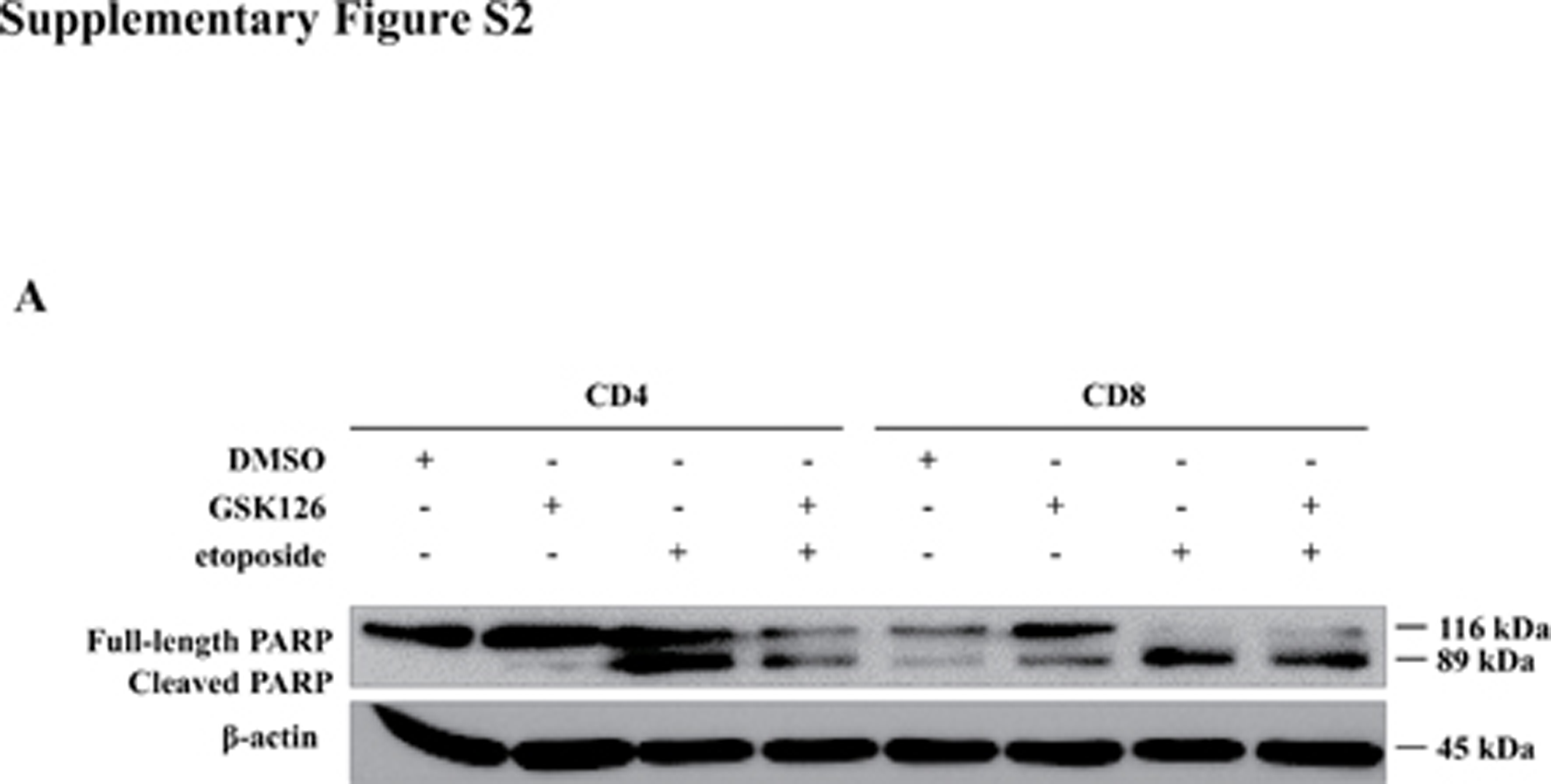

Supplement: Supplementary Figure 2 [file cddis2016198x3.tif]

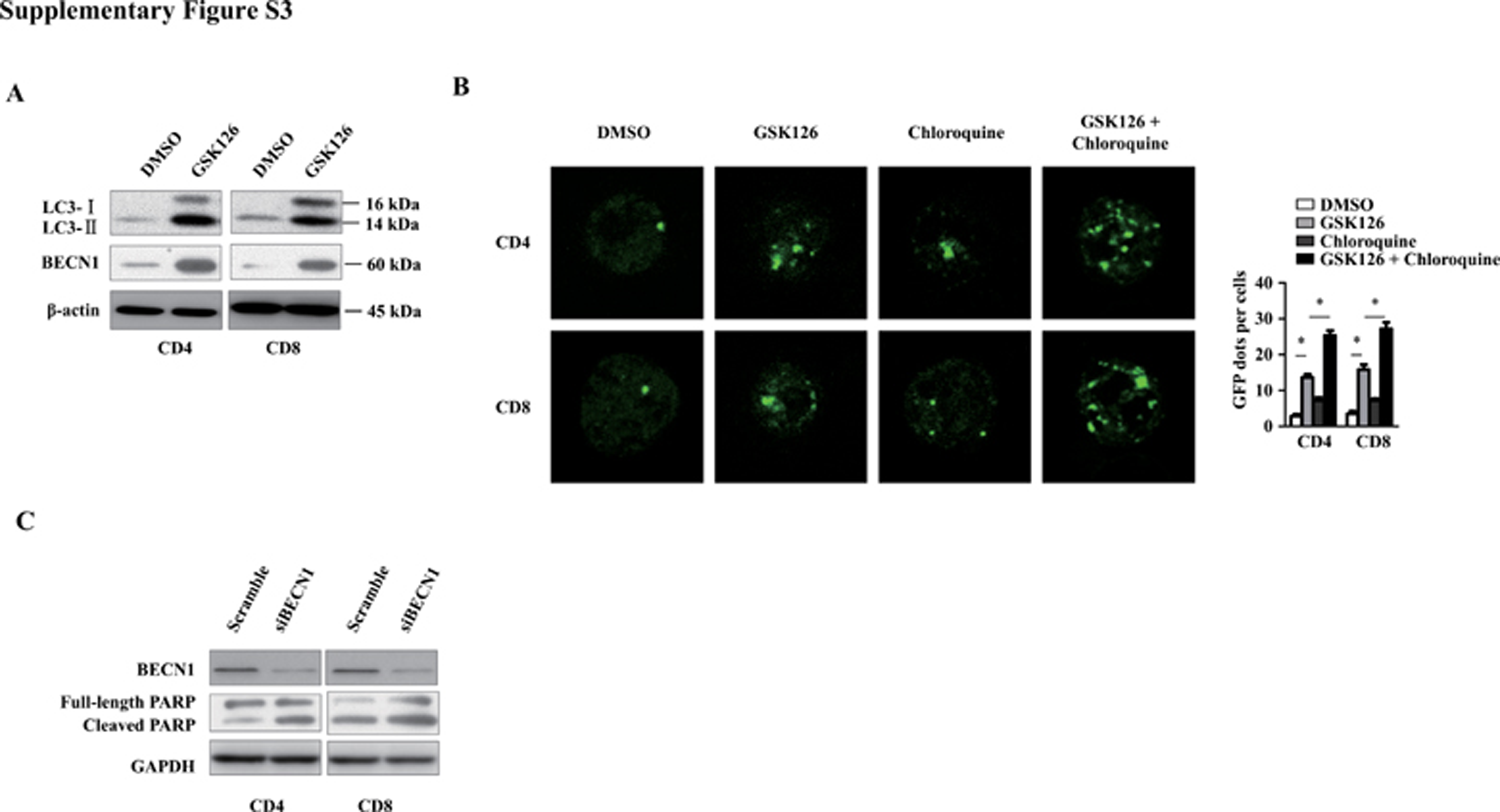

Supplement: Supplementary Figure 3 [file cddis2016198x4.tif]
