# Supplementary material for: DNA-PK-mediated phosphorylation of EZH2 regulates the DNA damage-induced apoptosis to maintain T-cell genomic integrity
Source: Cell Death Dis. 2016 Jul 28;7(7):e2316–. doi: 10.1038/cddis.2016.198 (PMC4973345; doi:10.1038/cddis.2016.198)
Supplement: Supplementary Tables [file cddis2016198x5.docx]

**Table S1. Specific primers used in Real-time PCR analysis**

| Gene | Primer | Sequence (5*'*→3*'*) |
| --- | --- | --- |
| *Eomes* | FW | AGACCAAATCGGAGCTGGGATCTC |
|  | RV | CTCAGAGAACTCGGGCTGACATCCT |
| *Bim* | FW | AGTGATTGGGCGTAGGAGCGG |
|  | RV | CTCCAACAAACTGCAGACCAGGC |
| *DAB2IP* | FW | ACATCACTCGCAGTGGC |
|  | RV | GGCGAGAGATATGGTTC |
| *MYT-1* | FW | ACAAAGGCAGATACCCAACG |
|  | RV | GCAGTTTCAAAAAGCCATCC |
| *GAPDH* | FW | TACTAGCGGTTTTACGGGCG |
|  | RV | TCGAACAGGAGGAGCAGAGAGCGA |

**Table S2. Specific primers used in CHIP analysis**

| Gene | Primer | Sequence (5*'*→3*'*) |
| --- | --- | --- |
| *Eomes* | FW | GCCATGCTTAGTGACACCGA |
|  | RV | GGACTGGAGGTAGTACCGC |
| *Bim* | FW | TCAGGGCTAGGGCCAGGCAG |
|  | RV | TATGGGTGGGGCGTGGGGAG |
| *DAB2IP* | FW | TGGACGATGTGCTCTATGCC |
|  | RV | GGATGGTGATGGTTTGGTAG |
| *MYT-1* | FW | ACTCCAGGCACCGAAGTTTAC |
|  | RV | AGAGGCGTCCTTCACCTCA |
| *GAPDH* | FW | TGACTCAACACGGGAAACCTCAC |
|  | RV | GGACATCTAAGGGCATCACAGACC |
